# Frequency of HIV Infection Among Pregnant Women in a Tertiary Care Hospital in Islamabad, Pakistan

**DOI:** 10.7759/cureus.79867

**Published:** 2025-03-01

**Authors:** Mishal Maqbool, Naushin Farooq, Laila Khalid, Qurrat ul Ain, Sabeen Aslam, Khadija Iftikhar, Lubna Saleem, Saadia Zia, Tehmina Kanwal

**Affiliations:** 1 Department of Obstetrics and Gynaecology, Federal Government Polyclinic Hospital, Islamabad, PAK; 2 Department of Hematology, Federal Government Polyclinic Hospital, Islamabad, PAK

**Keywords:** acquired immune deficiency syndrome (aids), high risk pregnancy, hiv aids, mother-to-child transmission, tertiary health care

## Abstract

Background

The prevalence of HIV among pregnant women remains a significant public health concern in Pakistan. Understanding the risk factors associated with HIV infection in this population is crucial for developing effective interventions and reducing the incidence of mother-to-child transmission (MTCT) of the virus.

Aim

This study aimed to assess the frequency of HIV infection among pregnant women attending a tertiary care hospital in Islamabad, Pakistan, and to identify the associated risk factors.

Methods

A cross-sectional study was conducted involving 130 pregnant women who presented with one or more HIV risk factors. Ethical approval and informed consent were obtained prior to the study. Participants underwent HIV testing, and data on demographics and HIV-related knowledge were collected. Statistical analysis was performed using IBM SPSS Statistics, utilizing descriptive statistics and chi-square tests to determine associations between HIV infection and various risk factors, with a significance threshold set at p < 0.05.

Results

Out of the 130 participants, 128 (98.5%) tested negative for HIV and 2 (1.5%) tested positive. The study revealed a low prevalence of unprotected sexual intercourse and a limited history of sexually transmitted infections (5.3%). Additionally, 97.7% of women reported limited knowledge about HIV transmission, and a small percentage reported barriers to accessing healthcare services, including HIV counseling.

Conclusion

The study found a low frequency of HIV among pregnant women in the sample population; however, it highlighted a significant lack of awareness regarding HIV transmission and related health services. Increased educational initiatives and accessible healthcare resources are recommended to enhance knowledge and reduce the risk of HIV among pregnant women, thereby minimizing the potential for MTCT.

## Introduction

HIV is a grave public health concern, still affecting a significant population worldwide. Globally, approximately 38 million people are living with HIV and AIDS, of whom 1.7 million contracted the virus for the first time in the year 2019 [1]. Approximately, one-half of this population includes women and children. There are 1.8 million affected children under 15 years of age and more than 17 million women [2]. Young undiagnosed women with HIV are at the risk of transmitting the virus to their offspring.

Prenatal HIV testing is necessary to identify HIV-positive women who should begin antiretroviral therapy (ARTs) to improve maternal health and reduce mother-to-child transmission (MTCT) [3]. Women who test negative for HIV during prenatal screening as part of this process might feel comforted in knowing that neither they nor their unborn children are at risk for HIV [4]. However, HIV can be acquired during pregnancy and the postpartum period. Thus, a repeat test is necessary, otherwise it could become a missed opportunity to start ARV treatment to prevent mother-to-child HIV transmission [5]. Although the frequency of MTCT during pregnancy, delivery, and breastfeeding is difficult to determine, some research suggested that more than half of the transmission has happened late in pregnancy or during labor and delivery [6].

The Centers for Disease Control and Prevention, along with other worldwide public health organizations, continue to prioritize the elimination of HIV's MTCT [7]. It is possible to lower the risk of MTCT of HIV from 25% to less than 1% with complete prenatal HIV care. Prenatal education regarding HIV status is necessary for putting preventative measures into practice [8]. A recent study found that 55.9% (562/1,005) women knew about MTCT, but only 30.3% (305/1,005) women had been tested [9]. In another study from Ethiopia, 57.5% of expectant mothers attending antenatal care services were fully aware of HIV's MTCT, whereas only 17.4% were aware of HIV/AIDS' MTCT. Women's lower levels of education were linked to less acceptance of HIV testing during pregnancy [10]. To improve understanding of the disease profile and facilitate the creation of HIV preventive and care initiatives, sociodemographic data on HIV patients are crucial [11].

The status of HIV infection among pregnant females in Pakistan shows a relatively low prevalence [12]. A study from Lahore reported that 0.35% of pregnant women were HIV-positive, and most of them never attended school or had a formal job [13]. Despite several HIV sub-epidemics in Larkana, HIV infection rate among pregnant women has remained low [14]. The overall HIV epidemic in Pakistan is concentrated in key populations such as people who inject drugs, males, females, and transgender sex workers [12]. Virus is also found in female spouses of HIV-positive injection drug users and bisexual men, as well as preadolescent children [15]. Efforts to combat HIV in Pakistan include regulating healthcare practices, promoting psychological counseling for HIV patients, educating the society, and minimizing commercial sex practices [16]. This study aimed to assess the frequency of HIV infection among pregnant females attending a tertiary care hospital in Islamabad.

## Materials and methods

Study design and data collection

In this cross-sectional research, the frequency of HIV infection among pregnant females attending Federal Government Polyclinic Hospital, Islamabad, Pakistan, was assessed. The sample size was 130, which was measured based on a confidence level of 95% and a margin of error of 1%. Pregnant women of all ages were included if they exhibited one or more of the risk factors. Pregnant females with known HIV-positive status before the study period or those with severe medical conditions or complications that may hinder their ability to participate in the study or undergo HIV testing were excluded. This focused approach aimed to identify individuals with a higher likelihood of infection.

Data analysis

The primary outcome was the frequency of HIV and associated risk factors among pregnant females. The hospital-based prevalence rates and identified risk factors were assessed. The data analysis was conducted using IBM SPSS Statistics Version 26 (IBM Corp., Armonk, NY). First, descriptive statistics was employed to summarize the demographic characteristics. Frequencies and percentages for categorical variables and means and standard deviations for continuous variables were measured. To assess the association between various risk factors and HIV infection, chi-square tests or Fisher's exact tests or t-tests were performed. A p-value of less than 0.05 was considered significant.

Ethical considerations

Ethical approval was taken from the Institutional Review Board. The consent process emphasized participants' rights, including the ability to withdraw from the study at any time without consequences, the confidentiality of personal information, and a complete explanation of the study's aims. Individuals presenting with any single risk factor were referred to Pakistan Institute of Medical Sciences (PIMS), Islamabad, Pakistan, for comprehensive evaluation. At PIMS, additional data such as clinical and demographic information were collected as part of the referral process.

## Results

In this study, a total of 130 pregnant women participated, with ages ranging from 17 to 45 years and a mean age of 28.5 years. The gestational age at enrollment ranged from 16 to 23 weeks, with a mean of 22.4 weeks. The majority of participants 92.3% were multigravida and 53.1% were primipara. A significant proportion of participants (97.6%) were in their first marriage, while the remaining were in their second marriage (Table 1).

**Table 1:** Baseline and gestational characteristics of patients (n=130)

Characteristic	No. of Cases	Percent
Age categories (years)		
Up to 25	54	41.5%
26 to 35	48	36.9%
35 or above	28	21.5%
Age (mean ± SD)	28.53± 7.71	
Gestational age (mean ± SD)	22.4 ± 4.2	
Parity		
Primipara	69	53.0%
Multipara	51	39.2%
Gravidity		
Primigravida	10	7.6%
Multigravida	120	92.3%
Marriage		
First	127	97.6%
Second	3	2.3%
History of IV drug use		
Yes	0	0.0%
No	130	100%
Unprotected sexual intercourse with multiple partners		
Yes	3	2.3%
No	127	97.6%
History of sexually transmitted infections		
Yes	7	5.3%
No	0	0.0%
Don’t know	123	94.6%
Presence of genital ulcers		
Yes	0	0.0%
No	130	100.0%
Previous pregnancies with HIV-positive status		
Yes	0	0%
No	130	100%

HIV testing showed that 128 (98.5%) participants tested negative and 2 (1.5%) tested positive. Analyzing the risk factors indicated that none of the participants acknowledged unprotected sexual intercourse with multiple partners. Also, 5.3% of cases reported a history of sexually transmitted infections (STIs), while 94.6% were uncertain about their STI history. None (0.0%) of participants reported genital ulcers and previous pregnancies with HIV-positive status (Table 1).

During the current pregnancy, 3% of participants reported limited access to healthcare, while 4.6% reported a lack of access to HIV counseling services. None of the participants reported migration or displacement from high-prevalence regions. Furthermore, 97.7% of participants reported limited knowledge about HIV transmission. A small proportion of participants reported histories of tuberculosis (1.54%) and malaria (2.31%), and 0.77% of participants reported hepatitis B and 2.31% reported hepatitis C.

Overall, 97% of cases reported partner-related risk factors, which included unknown HIV status and 2.31% of participants reported that their partners lived abroad. Histories of blood transfusion and dental procedures were reported by 0.77% and 1.5%, respectively (Table 2). Previous surgeries were recorded in 81.54%. Among the two HIV-positive participants, key shared risk factors were a history of prior surgery, the partner’s unknown HIV status, and limited knowledge about HIV (Table 3).

**Table 2:** Status on presentation and habits of study patients

Characteristic	No. of Cases	Percent
Sexual assault or coercion		
Yes	0	0%
No	130	100%
Lack of access to healthcare in the present pregnancy		
Yes	4	3.0%
No	126	96.9%
Lack of access to HIV counseling services		
Yes	6	4.6%
No	124	95.3%
Substance abuse including alcohol or illicit drugs		
Yes	0	0%
No	130	100%
Migration or displacement from regions with high HIV prevalence		
Yes	0	0%
No	130	100%
Limited knowledge about HIV transmission and prevention methods		
Yes	127	97.6%
No	3	2.3%
Any previous or current history of tuberculosis		
Yes	2	1.5%
No	122	93.8%
Any previous or current history of hepatitis B		
Yes	1	0.7%
No	124	95.3%
Any previous or current history of hepatitis C		
Yes	3	2.3%
No	122	93.8%
Any previous or current history of malaria		
Yes	3	2.3%
No	124	93.8%
Partner's HIV status		
Positive	0	0%
Negative	4	3.1%
Husband living abroad		
Yes	3	2.3%
No	127	97.6%
Sharing needles or syringes for medical purposes		
Yes	0	0.0%
No	130	100.0%
History of blood transfusion or organ transplantation		
Yes	1	0.7%
No	129	99.2%
History of any dental procedure		
Yes	2	1.5%
No	128	98.4%
History of any previous surgery		
Yes	106	81.5%
No	24	18.4%

**Table 3:** Association of baseline and gestational characteristics of patients with HIV status

Characteristic	Positive (n=2)	Negative (n=128)	p-Value
Age categories (years)			
Up to 25	2 (100.0%)	52 (40.6%)	0.239
26 to 35	0 (0.0%)	48 (37.5%)	
35 or above	0 (0.0%)	28 (21.8%)	
Age (mean ± SD)	19.0 ± 0.0	28.6 ± 7.6	<0.001
Gestational age (mean ± SD)	23.5 ± 2.1	22.4 ± 4.2	0.612
Parity			
Primipara	1 (50.0%)	68 (53.1%)	1.0
Multipara	1 (50.0%)	50 (39.0%)	
Gravidity			
Primigravida	0 (0.0%)	10 (7.8%)	1.0
Multigravida	2 (100.0%)	118 (92.1%)	
Marriage			
First	2 (100.0%)	125 (97.6%)	1.0
Second	0 (0.0%)	3 (2.3%)	
History of IV drug use			
Yes	0 (0.0%)	0 (0.0%)	1.0
No	2 (100.0%)	128 (100.0%)	
Unprotected sexual intercourse			
Yes	0 (0.0%)	8 (6.25%)	1.0
No	2 (100.0%)	128 (100.0%)	
History of STIs			
Yes	0 (0.0%)	7 (5.4%)	1.0
No	0 (0.0%)	0 (0.0%)	
Don’t know	2 (100.0%)	121 (94.5%)	
Presence of genital ulcers			
Yes	0 (0.0%)	0 (0.0%)	1.0
No	2 (100.0%)	128 (100.0%)	
Previous pregnancies with HIV-positive status			
Yes	0 (0.0%)	0 (0.0%)	1.0
No	2 (100.0%)	128 (100.0%)	

Despite the presence of risk factors at a critical level, these results align with the low prevalence of HIV within this cohort, but they also highlight the need to address gaps in knowledge, healthcare access, and spousal communication to mitigate future risks (Table 4).

**Table 4:** Association of presentation and habits of study patients with HIV status

Characteristic	Positive (n=2)	Negative (n=128)	p-Value
Sexual assault or coercion			
Yes	0 (0.0%)	0 (0.0%)	1.0
No	2 (100.0%)	128 (100.0%)	
Lack of access to healthcare			
Yes	1 (50.0%)	3 (2.3%)	0.061
No	1 (50.0%)	125 (97.6%)	
Lack of access to HIV counseling			
Yes	0 (0.0%)	6 (4.6%)	1.0
No	2 (100.0%)	122 (95.3%)	
Substance abuse (alcohol or drugs)			
Yes	0 (0.0%)	0 (0.0%)	1.0
No	2 (100.0%)	128 (100.0%)	
Migration from high HIV prevalence regions			
Yes	0 (0.0%)	0 (0.0%)	1.0
No	2 (100.0%)	128 (100.0%)	
Limited knowledge about HIV			
Yes	2(100.0%)	125(97.6%)	1.0
No	0 (0.0%)	3 (2.3%)	
History of tuberculosis			
Yes	0 (0.0%)	2(1.5%)	1.0
No	2 (100.0%)	120(93.7%)	
History of hepatitis B			
Yes	1 (50.0%)	0 (0.0%)	0.016
No	1(50.0%)	123 (96.1%)	
History of hepatitis C			
Yes	1 (50.0%)	2 (1.5%)	0.048
No	1 (50.0%)	121 (94.5%)	
History of malaria			
Yes	0 (0.0%)	3 (2.3%)	1.0
No	2 (100.0%)	122 (95.3%)	
Partner's HIV status			
Positive	0 (0.0%)	0 (0.0%)	1.0
Negative	0 (0.0%)	4 (3.1%)	
Husband living abroad			
Yes	1 (50.0%)	2(1.5%)	0.046
No	1 (50.0%)	126 (98.4%)	
Sharing needles for medical purposes			
Yes	0 (0.0%)	0 (0.0%)	1.0
No	2 (100.0%)	128 (100.0%)	
History of blood transfusion			
Yes	0 (0.0%)	1 (0.7%)	1.0
No	2 (100.0%)	127 (99.2%)	
History of dental procedures			
Yes	0 (0.0%)	0 (0.0%)	1.0
No	2 (100.0%)	128 (100.0%)	
History of previous surgery			
Yes	2 (100.0%)	104 (81.2%)	1.0
No	0 (0.0%)	24 (18.7%)	

## Discussion

This study reported an HIV prevalence of 1.5% among pregnant women attending a tertiary care hospital in Islamabad, Pakistan, which aligns with previous local studies. In Karachi, research was conducted, which found a prevalence of 1.2%, which reflects the relatively low rates of HIV in pregnant women in Pakistan [17]. Despite this apparently low prevalence, this is a worrisome situation. This study highlights risk factors such as partner’s unknown HIV status, a history of STIs, and lack of health education and access to healthcare at a critical level. Our findings indicate that all HIV-positive cases belonged to the youngest age group (≤25 years), which suggests that younger women may be more vulnerable due to factors such as limited awareness and reduced access to healthcare services. This shows the need for special efforts to stop the spread.

There were 2.3% of participants who reported unprotected sexual intercourse, which is also a significant risk factor for HIV transmission. A study conducted in Lahore similarly identified unprotected sexual practices as a key driver of HIV among women especially in the context of partner-related risks such as extramarital relationships [18]. However, in our study, none of the HIV-positive participants reported unprotected intercourse, which suggests that other risk factors may have played a role in transmission. There were 127 (97.6%) participants who reported limited knowledge about HIV knowledge and transmission and prevention, which may emerge as a significant risk factor for HIV transmission. The cultural norms in South Asia limit the discussion and communication around sexual health, which hinders education about HIV transmission and prevention [19]. To address these issues, there is a need for comprehensive sexual education and community programs to promote safer habits and reduce shame about HIV testing.

Most of the study participants, 123 (96.0%), were unaware of their history of STIs. This finding is significant because STIs are a known risk factor for HIV, and affected women experience increased vulnerability due to compromised mucosal integrity, which facilitates viral entry. In a study conducted in Karachi, women with untreated STIs were at a higher risk of HIV acquisition [20]. This result matches studies from other countries, such as the one conducted in South Africa, which clearly showed how STIs and HIV are connected and affect each other [21]. In our study, both HIV-positive participants did not know their STI history, which further highlights the gap in STI diagnosis and awareness in antenatal populations. Therefore, it is necessary to integrate STI screening and treatment into routine antenatal care services in Pakistan to mitigate this dual risk.

Only four participants reported having limited access to healthcare. However, HIV counseling services remain severely limited across the country, indicating broader systemic gaps in HIV-related support. A study from Khyber Pakhtunkhwa reported that due to geographic and socioeconomic barriers, there is limited access to antenatal care, thus delaying HIV diagnosis and treatment [22]. Similar patterns have been observed globally, especially in low-resource settings, where such barriers increase the risk of MTCT [23]. Improving the healthcare facilities and fixing unfair differences in the system are very important to help reduce HIV problems in Pakistan. Our study found that 50% of HIV-positive participants had reported limited access to healthcare, which highlights that a lack of healthcare access remains a key factor in HIV transmission.

Migrant populations in Pakistan, particularly those returning from high-prevalence regions such as the Middle East, face heightened risks. Moreover, due to internal conflicts, the internally displaced populations are also vulnerable. Limited healthcare access and greater exposure to high-risk behaviors further increase their susceptibility [24]. In Punjab, a study was conducted, which highlighted that many returning workers were unaware of their HIV status, which underscores the need for targeted strategies for this vulnerable group [25]. To manage these risks, health checkups are important after returning home. Although none of our HIV-positive participants had a history of migration, it remains an important risk factor to be considered in future research.

A critical concern is MTCT, with an estimated risk of 15-45% without intervention. However, this risk can be reduced to below 5% with effective ART and obstetric management. A recent study in Rawalpindi showed the effectiveness of routine antenatal HIV screening and ART in reducing MTCT rates to negligible levels [19]. The World Health Organization stresses the need for HIV testing during pregnancy to eliminate the spread of HIV from mother to child [26]. In our study, none of the HIV-positive participants had previous pregnancies with HIV, which suggests that timely diagnosis in first pregnancies is crucial to prevent MTCT in subsequent pregnancies.

This study highlights the challenges in HIV prevention among pregnant women both regionally and globally. The rates of HIV in pregnancy in Pakistan show a window of opportunity to curtail index transmission. However, there are still major gaps in education, healthcare access, and involvement of male partners. Furthermore, our study found significant associations between HIV status and hepatitis infections as well as husbands living abroad, which suggests that in future studies these factors should be explored further. To provide a deeper understanding, there is a need for future studies in Pakistan with larger and more diverse samples.

## Conclusions

The frequency of HIV infection in pregnant women was 1.5%. Despite this apparently low prevalence, this study highlights the risk of MTCT. History of STIs, limited access to healthcare and health education, and partner’s unknown HIV status were some of the probable factors. The results also emphasize the importance of sexual education programs, STI screening and treatment, and improving healthcare access to mitigate the risk of HIV transmission. This study also adds to the understanding of HIV prevention in pregnant women in Pakistan.
